# Dendritic Cells in Dengue Virus Infection: Targets of Virus Replication and Mediators of Immunity

**DOI:** 10.3389/fimmu.2014.00647

**Published:** 2014-12-17

**Authors:** Michael A. Schmid, Michael S. Diamond, Eva Harris

**Affiliations:** ^1^Division of Infectious Diseases and Vaccinology, School of Public Health, University of California Berkeley, Berkeley, CA, USA; ^2^Department of Medicine, Washington University School of Medicine, St. Louis, MO, USA; ^3^Department of Molecular Microbiology, Washington University School of Medicine, St. Louis, MO, USA; ^4^Department of Pathology and Immunology, Washington University School of Medicine, St. Louis, MO, USA

**Keywords:** dengue virus, dendritic cells, monocytes, macrophages, innate immunity, antibody-dependent enhancement, immune evasion

## Abstract

Dendritic cells (DCs) are sentinels of the immune system and detect pathogens at sites of entry, such as the skin. In addition to the ability of DCs to control infections directly via their innate immune functions, DCs help to prime adaptive B- and T-cell responses by processing and presenting antigen in lymphoid tissues. Infected *Aedes aegypti* or *Aedes albopictus* mosquitoes transmit the four dengue virus (DENV) serotypes to humans while probing for small blood vessels in the skin. DENV causes the most prevalent arthropod-borne viral disease in humans, yet no vaccine or specific therapeutic is currently licensed. Although primary DENV infection confers life-long protective immunity against re-infection with the same DENV serotype, secondary infection with a different DENV serotype can lead to increased disease severity via cross-reactive T-cells or enhancing antibodies. This review summarizes recent findings in humans and animal models about DENV infection of DCs, monocytes, and macrophages. We discuss the dual role of DCs as both targets of DENV replication and mediators of innate and adaptive immunity, and summarize immune evasion strategies whereby DENV impairs the function of infected DCs. We suggest that DCs play a key role in priming DENV-specific neutralizing or potentially harmful memory B- and T-cell responses, and that future DC-directed therapies may help induce protective memory responses and reduce dengue pathogenesis.

## Introduction

### Dendritic cells, monocytes, and macrophages: Location and function

Dendritic cells (DCs) reside and migrate into barrier tissues such as the skin and mucosal epithelium that are the sites of pathogen invasion. In the steady state, DCs display high levels of phagocytic activity, take up antigen, and probe for pathogens via pattern-recognition receptors. DCs express Toll-like receptors (TLRs) and C-type lectins as transmembrane proteins as well as intracellular sensors, such as retinoic acid-inducible gene I (RIG-I), melanoma differentiation factor 5 (MDA-5), and cyclic GMP-AMP synthase (cGAS) that recognize conserved microbial patterns (1, 2). Upon pathogen recognition, DCs become activated, produce inflammatory cytokines and chemokines, migrate to lymph nodes (LNs), and present antigen to prime naïve T-cells (3).

Subsets of DCs, monocytes, and macrophages (MΦs) reside in different tissues and fulfill distinct functions. Classical DCs (cDCs) display the characteristic DC dendrites, are present in LNs, spleen, and bone marrow as well as skin, lung, liver, and intestine, and have the greatest ability to stimulate naïve T-cells (4). All cDCs can present endogenous antigen from the cytosol via MHC I to CD8^+^ T-cells (5). In addition, most subsets of cDCs present exogenous antigen via MHC II to CD4^+^ T-cells, whereas only specialized subsets can cross-present exogenous antigen via MHC I to CD8^+^ T-cells (6, 7). In addition, plasmacytoid DCs (pDCs) are another DC subset that reside in the spleen, bone marrow, and liver and circulate in the blood. During viral infections, pDCs migrate to infected tissues and secrete up to 1,000-fold higher amounts of interferon (IFN)-α/β than other cell types (8), although their capacity for antigen presentation is still debated (9). Nevertheless, the role of DCs in priming protective immune responses against many human pathogens and their potential contribution to pathogenesis and development of disease need further investigation.

Monocytes circulate in steady-state blood, patrol lymphoid, and non-lymphoid organs, and are recruited to inflamed tissues, where they phagocytize pathogens as well as infected or damaged cells (10). During inflammation, monocytes can differentiate to monocyte-derived DCs (moDCs) (11, 12). *In vitro*-generated human moDCs are used widely to study DC biology (13). Monocytes are isolated from human peripheral blood, differentiated in the presence of GM-CSF and IL-4 first to immature moDCs and after further stimulation with inflammatory cytokines or pathogen-associated microbial patterns (PAMPs) to mature moDCs (13, 14). The ability of moDCs to prime naïve T-cell responses remains controversial, as this function initially was attributed solely to cDCs (15). Nevertheless, recent studies demonstrated that moDCs can migrate to LNs and prime naïve T-cells during *Leishmania major*, influenza virus, and bacterial infections (12, 16, 17). In contrast to DCs, MΦs have limited ability to migrate and prime naïve T-cells. MΦs reside within tissues, where they phagocytize, secrete cytokines, present antigen to effector and memory T-cells, and contribute to the healing of injured tissue (18).

### Dengue epidemiology and pathogenesis

Female *Aedes aegypti* and *Aedes albopictus* mosquitoes transmit the four dengue virus serotypes (DENV1–4) while feeding on blood vessels in the skin (19). The positive-sense RNA genome of the flavivirus DENV encodes three structural (C, prM/M, E) and seven non-structural (NS) proteins (20). DENV causes the most prevalent arthropod-borne viral disease of humans, with an estimated 390 million infections and 96 million apparent cases per year (21).

The acute febrile illness dengue fever (DF) can progress to a potentially life-threatening vascular leakage syndrome, dengue hemorrhagic fever/dengue shock syndrome (DHF/DSS), the latter characterized by hypotension and circulatory failure (22). At present, no vaccine or therapeutic against dengue is approved for use in humans. A major challenge in the development of vaccines and therapies is that although infection with one DENV serotype leads to long-lasting immunity against the same serotype, subsequent infection with a different (heterotypic) serotype is the major risk factor for severe disease (19). To date, the mechanisms by which the host immune response to DENV provides either protection or enhancement in secondary infection remain poorly understood. Antibodies can neutralize infection or conversely trigger “antibody-dependent enhancement” (ADE) (23, 24), whereby cross-reactive anti-DENV antibodies facilitate entry of DENV into Fcγ receptor (FcγR)-bearing cells and thus increase viral load and ultimately disease severity. Some DHF/DSS cases occur during primary (1°) infections, especially in infants 6–9 months of age (25). In this case, it is thought that maternal DENV-specific antibodies transferred via the placenta wane to levels that can enhance a newly acquired DENV infection (26). Thus, the quantity and quality of the antibody response influences the severity of a secondary DENV infection. Similarly, T-cells can provide protection (27–29), but cross-reactive T-cells have been implicated in disease pathogenesis (30–32). Nevertheless, most secondary DENV infections are asymptomatic or mild, suggesting that the immune system can mount protective responses against dengue.

*Aedes* mosquitoes that take a blood meal from a human with acute dengue viremia become infected and, after DENV spreads to the salivary glands, transmit the virus when feeding on a new individual. Mosquito saliva contains components that counteract the host hemostatic response and modulate immunity (33, 34). The addition of saliva from *Ae. aegypti* mosquitoes was found to decrease DENV infection of moDCs *in vitro* (35). In contrast, mosquito saliva or transmission via infected mosquitoes prolonged DENV serum viremia and fever in “humanized” mice as compared to inoculation with DENV alone (36). Furthermore, saliva that was inoculated by non-infected mosquitoes prior to needle inoculation of DENV blocked the upregulation of genes involved in innate pathogen recognition and increased serum viremia in mice deficient in IRF3 and IRF7 (37). Although certain *in vivo* studies suggest that mosquito saliva can facilitate DENV infection by generating an environment that favors early virus replication, the impact of saliva on skin DCs requires further study.

## Targets of DENV Replication

### DENV infection in the absence of enhancing antibodies

Identifying the targets of DENV infection is crucial for understanding virus spread and disease pathogenesis. Human autopsies revealed staining for DENV structural proteins and negative-sense viral RNA, indicative of virus replication, in MΦs in LNs, spleen, lung, and liver and monocytes in clotted blood from patients with lethal dengue disease (38, 39). Staining of the non-structural protein NS3 confirmed DENV replication in phagocytes (including monocytes, MΦs, and DCs) in LNs and spleen, as well as in MΦs in the lung in other autopsy studies (40, 41). In earlier stages of the disease, most DENV-infected cells in the peripheral blood of acute dengue patients were identified as CD14^+^ CD11c^+^ activated monocytes, with higher proportions of monocytes and DENV-infected total cells in the blood in DHF compared to DF patients (42).

Although in humans, DENV efficiently suppresses the IFN response, replicates, and causes disease, DENV fails to antagonize mouse IFN responses, and thus wild-type (WT) mice generally do not sustain DENV replication or develop disease (43). In comparison, mice deficient in IFN-α/β receptor (*Ifnar*^−/−^) and also -γ receptor (AG129) are susceptible to DENV infection and display a tropism similar to humans (40, 44–46). DENV replicated in murine MΦs that were isolated from the peritoneum (47), as well as in MΦs in LNs and spleens of AG129 mice (48, 49). Treatment with clodronate liposomes that deplete monocytes and MΦs decreased viral load in AG129 mice on day 2 but increased viral load on day 4 post-inoculation with DENV2 (48). Monocytes and MΦs thus play an important role as targets for early DENV replication as well as in subsequent control of DENV infection.

Human moDCs generated *in vitro* support DENV infection (50), with immature moDCs being more susceptible to DENV infection than mature moDCs, monocytes (51), or MΦs (52). Analogously, CD11c^high^ cells in the spleen of AG129 mice (47) that likely comprised both moDCs and cDCs supported DENV replication *in vivo*. Recent studies in *Ifnar*^−/−^ mice have shown that early after infection, monocytes are recruited to the dermis and differentiate to moDCs, where they become primary targets for DENV replication (53). Although DENV can infect monocytes and MΦs directly, these studies emphasize the greater permissiveness of DCs to DENV infection in the absence of enhancing antibodies, such as during 1° infection conditions.

**Figure 1:**
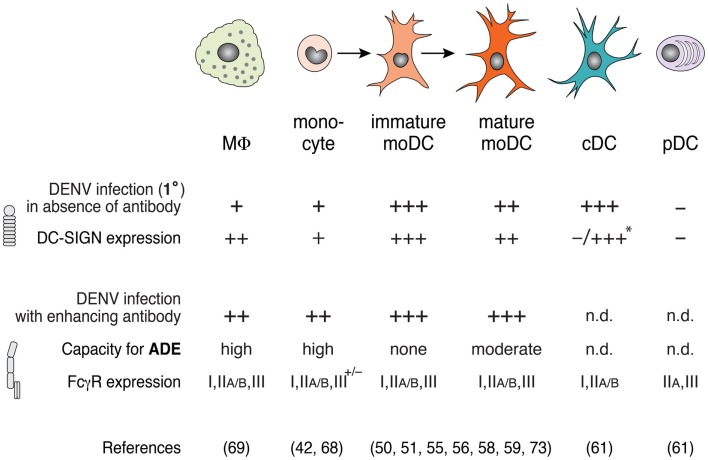
**DENV infection varies among macrophages, monocyte, and dendritic cell (DC) subsets in the presence or absence of enhancing antibodies**. DC-SIGN expression correlates with high infection in the absence of enhancing antibodies (i.e., 1° infection conditions), whereas FcγR expression modulates antibody-enhanced infection during ADE. Macrophages (MΦ) and monocytes express low levels of DC-SIGN, and show little DENV infection in the absence of enhancing antibody, but are highly infected in the presence of enhancing antibody. Under inflammatory conditions, monocytes differentiate to immature monocyte-derived DCs (moDCs) and, further, to mature moDCs after stimulation via PAMPs or inflammatory cytokines. While immature moDCs express high levels of DC-SIGN and can be infected with DENV in the absence of antibodies, mature moDCs express lower levels of DC-SIGN and show moderate permissiveness under these conditions. Accordingly, mature moDCs show a capacity for enhanced infection in the presence of subneutralizing anti-DENV antibodies. Classical DCs (cDCs) that are freshly isolated from human blood do not express DC-SIGN, but express high levels of DC-SIGN after stimulation with GM-CSF and IL-4 *in vitro* (*), which renders them highly susceptible to DENV infection without antibody, similar to immature moDCs. Plasmacytoid DCs (pDCs) do not express DC-SIGN or support DENV replication in the absence of antibody. cDCs and pDCs express FcγRs, but DENV infection of cDCs and pDCs during ADE has not been determined (n.d.).

Surface expression of viral attachment factors determines the susceptibility to DENV infection. DC-SIGN (dendritic-cell-specific ICAM3-grabbing non-integrin, CD209) is a C-type lectin expressed on the surface of DCs and MΦs that recognizes mannose-type sugars on the surface of bacterial, fungal, and viral pathogens. Signals via DC-SIGN induce the phagocytosis of pathogens and contribute to host defense (54). However, DC-SIGN also interacts with carbohydrates on DENV glycoproteins and mediates the attachment of DENV to moDCs (55–57). Human immature moDCs express high levels of DC-SIGN and are highly susceptible to DENV infection (50, 58, 59). Of note, DC-SIGN mediates virus attachment to the cell surface, but not endocytosis into moDCs (60). Activation of immature moDCs via inflammatory cytokines results in downregulation of DC-SIGN, explaining in part why mature moDCs are less susceptible to DENV infection (51, 58). Further, human cDCs freshly isolated from blood do not express DC-SIGN and, accordingly, become highly susceptible to DENV infection only after culture with GM-CSF and IL-4 that induces DC-SIGN expression (61). Similarly, treatment of monocytes with IL-4 or IL-13 increased DC-SIGN expression and DENV infection (62), but it remains unclear whether these monocytes had differentiated to moDCs or MΦs. Consistent with these findings, higher levels of DC-SIGN expression on cDCs of different blood donors correlated with higher DENV infection (61). Furthermore, a polymorphic variant of the DC-SIGN promoter with a decreased transcriptional activity correlated with protection against DF in humans (63). In summary, changes in DC-SIGN expression on different myeloid cell subsets through differentiation correlate with DENV infection (Figure 1).

Although *Aedes* mosquitoes transmit DENV when probing for blood vessels in the skin, most studies have focused on DENV infection in tissues after the virus has spread via the blood. Few studies have examined DENV infection and the immune response in the skin. DENV infects epidermal Langerhans cells (LCs) in healthy human skin explants *in vitro* (58, 64). Infection of LCs was confirmed in AG129 mice after intradermal inoculation of DENV2 (48). However, the dermis of intradermally inoculated *Ifnar*^−/−^ mice contains 100-fold more DENV-infected cells than the epidermis (53). Recent studies indicate that dermal cDCs, and to a lesser extent MΦs, are the initial targets of DENV replication after intradermal inoculation of *Ifnar*^−/−^ mice (53) or infection of skin explants from healthy human donors (65). Subsequently, *de novo*-recruited monocytes differentiate into moDCs, which become primary targets for DENV replication in the dermis (53).

Regarding the source of infectious virus, DENV produced in mosquito cells interacts with DC-SIGN and infected human immature moDCs *in vitro* (66). In contrast, DENV that was produced in human moDCs did not bind to DC-SIGN or infect moDCs but instead was infectious for cells expressing the homolog L-SIGN, such as monocytes and endothelial cells (66). The difference in binding to DC-SIGN or L-SIGN was likely due to different N-linked glycosylation patterns present on DENV particles produced in mosquito or mammalian cells. This may explain how DCs in the skin are the initial targets for DENV infection immediately after transmission. Characterization of the initial targets and immune response to DENV in the skin may foster new strategies to block DENV replication and abort pathogenesis.

### Antibody-enhanced DENV infection

Dengue virus-specific adaptive immune responses, in particular subneutralizing concentrations of antibodies, can enhance DENV entry and infection. In cell culture, subneutralizing amounts of DENV-immune human serum or monoclonal antibodies enhance infection of monocytes (67, 68) and mature moDCs, but not immature moDCs (51). Human splenic MΦs showed low levels of DENV infection at baseline *in vitro*, but at least 10-fold greater infection in the presence of enhancing concentrations of diluted DENV-immune human (69). *In vivo*, CD14^+^ monocytes in the blood of acute dengue patients contained significantly higher levels of DENV genomic RNA in severe DHF compared to DF cases, and in secondary compared to 1° infections (70). Thus, ADE mediates efficient DENV infection of monocytes, mature moDCs, and MΦs.

FcγR expression determines the susceptibility of cells to ADE during DENV infection via uptake of virus-antibody complexes (Figure 1). Most myeloid cells express FcγRs, which bind the Fc region of antibodies and thus are an important link between cellular effector functions and antigen recognition via the antibody. Different types of FcγRs recognize distinct isotypes of IgG with varying affinity and can transmit activating or inhibitory signals to the cells (71). Attachment of DENV-antibody complexes to ectopically expressed FcγRI (CD64) (72) and FcγRIIA (CD32) (73) in fibroblast cell lines mediated ADE, independently of FcγR-signaling. DENV2 infection in primary human monocytes was increased 50-fold in the presence of enhancing DENV-immune human serum and depended on binding of antibody-virus complexes to FcγRI or FcγRIIA (68). Consistent with these data, blocking of FcγRIIA, but not of FcγRIIB, abrogated ADE of mature moDCs (51). Although mature and immature moDCs express similar levels of FcγRIIA, only mature moDCs sustain antibody-enhanced DENV infection because immature moDCs express high levels of DC-SIGN and thus do not require FcγR for DENV attachment or entry (51). Inflammation may lead to activation and differentiation of monocytes and moDCs, as well as altered expression of FcγRs and DC-SIGN, which modulates ADE of DENV infection in a cell-type specific manner.

Different classes of FcγRs transmit activating or inhibitory signals and play different roles during ADE. Although activating FcγRIIA and inhibitory FcγRIIB similarly bind DENV-antibody complexes, only FcγRIIA mediates enhanced DENV infection (74). In contrast to activating FcγRs, the inhibitory FcγRIIB may help prevent ADE. DENV-antibody aggregates cross-linked the inhibitory low-affinity FcγRIIB, which inhibited the phagocytosis and infection that would have occurred through activating FcγRs (75). This study showed evidence that the size of antibody-DENV aggregates may contribute to the neutralizing versus enhancing capacity of DENV-immune sera. More detailed studies on the expression of activating and inhibitory FcγRs are needed to understand the neutralization or enhancement of DENV infection of diverse cell subsets in different tissues. Along with greater infection *in vitro*, ADE may contribute to severe dengue disease in humans. Secondary DENV infection with a heterologous serotype is associated with an increased risk of DHF/DSS (76–79). Consistent with a possible role for ADE *in vivo*, polymorphisms in FcγR genes affect binding affinities for IgG subclasses and may influence the susceptibility to severe disease. Homozygotes for the arginine variant at position 131 (R/R131) of the FcRIIA gene, who have less capacity to opsonize IgG_2_ antibodies, showed reduced risk of developing DHF (80, 81). In contrast, the histidine variant H/H131 of FcRIIA was associated with an increased risk of developing DHF (81). Binding affinity of FcγRs to DENV-antibody complexes and the ratio of activating and inhibitory receptors likely determine DENV infection and disease outcome in the setting of pre-existing anti-DENV antibody.

Animal models also have been used to study ADE *in vivo*. AG129 mice develop mild disease after intravenous or intradermal inoculation with DENV2 in the absence of pre-existing antibody and lethal disease after passive transfer of subneutralizing levels of DENV-immune mouse or human serum prior to infection with otherwise sublethal doses of DENV (53, 82–84). The vascular leakage syndrome that develops in DENV-infected AG129 mice during ADE is similar to that observed after high-dose lethal DENV infection and recapitulates many features of severe dengue disease in humans (40, 44). Monoclonal antibodies directed against DENV protein E or prM can mediate ADE *in vitro* and *in vivo* (82, 83, 85). Addition of antibodies that block FcγR binding, F(ab)’_2_ fragments that lack the Fc domain, or recombinant monoclonal antibodies that lack the ability to bind FcγR all prevented ADE (82, 83). DENV-specific monoclonal antibodies with modified Fc domains that do not mediate ADE show therapeutic potential *in vivo* (85–87).

Mouse models have helped to define possible cellular targets for antibody-enhanced DENV infection. While the same cell types become DENV infected in the presence or absence of enhancing antibodies, DENV infection increases during ADE (82). In addition, MHC II^+^ cells in the intestinal lamina propria and sinusoidal endothelial cells in the liver of AG129 mice were infected by DENV mostly in the presence of enhancing concentrations of antibody (83). More detailed studies using human cells *in vitro*, clinical samples, and animal models are needed to clarify how cellular activation and differentiation modulate FcγR expression and impacts DENV infection and pathogenesis.

## Innate Function of Dendritic Cells and Evasion by DENV

As first line of defense against virus infection, host cells recognize PAMPs (e.g., viral nucleic acids) and induce cell-intrinsic and cell-extrinsic innate immune responses. DENV infection stimulates responses via TLR7, TLR3, MDA5, and RIG-I (88–90) and induces the secretion of IFN-α/β that renders other host cells resistant to subsequent DENV infection (91, 92).

Plasmacytoid DCs recognize DENV via TLR7 in endocytic vesicles (61, 89), become activated, produce high amounts of IFN-α (93, 94), and may thus limit DENV replication. Further, pDCs sense DENV-infected cells by direct cell-to-cell contact, and immature DENV particles containing uncleaved prM were found to trigger higher IFN responses in pDCs compared to mature particles (95). Nevertheless, DENV does not infect human pDCs efficiently *in vitro*, and IFN-α production in pDCs appears independent of active viral replication within pDCs (61). This suggests that pDCs combat DENV without being susceptible to infection or to immune evasion mediated via cytosolic viral proteins. Because DENV cannot infect pDCs productively, its proteins cannot block the production of pDC-derived IFN-α that promotes transcription of IFN-stimulated genes (ISGs), which induce an antiviral state in neighboring cells. Patients with non-severe DF produced high levels of IFN-α in the serum (93, 96) and had an increased frequency of circulating pDCs compared to steady state (94). In contrast, numbers of pDCs declined early (day 3 or 4 of illness) in the blood of children who subsequently developed DHF (94) and, correspondingly, accumulated less in severe compared to non-severe adult cases (93). Furthermore, serum of patients with severe dengue contained less IFN-α than patients with non-severe manifestations (96). Consistent with this observation, peripheral blood mononuclear cells of patients that subsequently developed DSS expressed fewer transcripts of a set of ISGs than patients with mild disease (97, 98). Overall, pDCs protect against DENV pathogenesis by producing high amounts of IFN-α that prevent infection of additional target cells.

Dengue virus actively blocks the production and action of IFN-α/β in cell types that are susceptible to infection. The DENV non-structural protein NS2B/3 cleaves the human protein STING (also known as MITA) (99, 100), which is a key adaptor molecule in the cellular response to virus infection and in establishing the basal set-point of IRF3 signaling and IFN-α/β production (101). DENV NS5 protein induces targeted degradation of STAT2 via the proteasome (102–105) and NS4B blocks STAT1 activation (102, 106) and thus inhibits IFN-α/β and likely IFN-γ and IFN-λ receptor signaling in DENV-infected cells. However, non-infected cells remain capable of IFN production and IFNAR signaling, which may induce resistance to subsequent DENV infection. Indeed, pre-treatment of cells with IFN-α/β or IFN-γ prevents DENV infection (107–109).

Antibody-enhanced DENV infection also is believed to contribute to the evasion of the innate immune response through a mechanism termed “intrinsic ADE” (110). A monocytic cell line, THP-1, showed decreased production of inflammatory cytokines and mediators, such as IL-12, IFN-γ, TNFα, and nitric oxide radicals when they were infected with DENV in the presence but not in the absence of enhancing antibodies (111). Similarly, ADE infection suppressed TLR-mediated signals and the secretion of IFN-β, but increased the production of anti-inflammatory cytokines, such as IL-10, compared to DENV infection without antibodies (112). These effects depended on FcγR binding because antibodies blocking FcγRI or FcγRIIA restored IFN-β production (112). These findings were unexpected because activating FcγR signals should induce expression of ISGs and thus block DENV replication. Recent studies showed that the leukocyte immunoglobulin-like receptor-B1 binds DENV-antibody aggregates and blocks activating FcγR signals, which enabled DENV to evade the early antiviral response during ADE (113). These results suggest that antibody-mediated DENV entry also triggers intracellular signals that suppress innate responses in infected cells to increase viral production. This “intrinsic ADE” is complemented by “extrinsic ADE,” which refers to the enhanced FcγR-mediated binding and uptake of DENV described above.

## Priming and Evasion of Adaptive Immune Responses

Dendritic cells link innate and adaptive immune responses by integrating innate signals from PAMPs with pathogen-derived antigens to induce antigen-specific T-cell and B-cell responses. DCs achieve this by (a) taking up and processing antigen and presenting antigen-derived peptides on MHC I to CD8^+^ T-cells or on MHC II to CD4^+^ T-cells; (b) expressing co-stimulatory molecules that activate T-cells; and (c) secreting chemokines and cytokines that attract T-cells and modulate the priming T-cell effector functions.

Because DC survival is required for optimal T-cell activation, DENV-induced apoptosis of DCs could antagonize the priming of immune responses. Bulk culture studies observed increased survival of moDCs (50, 114) and monocytes (115) after exposure to DENV. However, intracellular staining studies revealed a higher fraction of Annexin V^+^ apoptotic cells in those co-staining for DENV E protein (116). These results suggest that DENV induces apoptosis in infected cells, but increases survival in non-infected bystander cells. The impact of cell survival on the number of antigen-presenting cells and priming of DENV-specific adaptive immune responses warrants further study.

**Figure 2:**
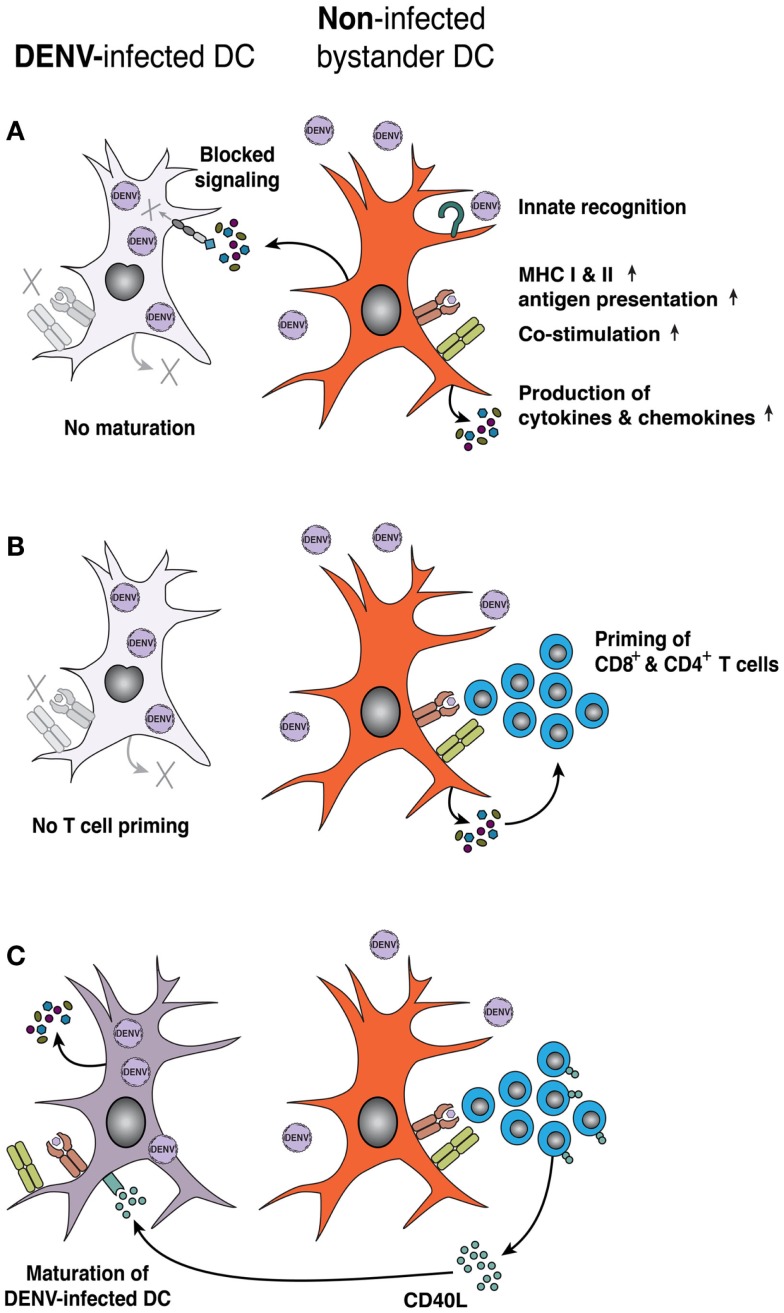
**DENV infection impairs DC activation and priming of adaptive T-cell responses**. **(A)** Maturation of human moDCs is inhibited by DENV infection, due to DENV non-structural proteins blocking induction and intracellular signaling of IFN-α/β. However, non-infected bystander DCs respond to PAMPs and/or cytokines associated with DENV infection and upregulate MHC class I and II molecules, co-stimulatory molecules, and the expression of inflammatory cytokines and chemokines. **(B)** Mature, bystander DCs efficiently prime adaptive T-cell responses, whereas DENV-infected DCs prime naïve T-cells less efficiently. **(C)** Activated T-cells display CD40L on their surface or secrete CD40L that acts on DENV-infected DCs and can restore DC maturation and function. Purple denotes DENV infection, while orange denotes inflammation/cell activation.

Pathogen recognition leads to DC maturation, which is characterized by increased expression of MHC II and co-stimulatory markers required for efficient priming of T-cell responses (117). After exposure to DENV, non-infected bystander moDCs upregulate MHC I and II molecules, as well as co-stimulatory molecules CD80 (B7-1), CD83, and CD86 (B7-2), although DENV blocks activation and maturation of infected moDCs within the same cultures (59, 116) (Figure 2A). Intracellular staining for DENV proteins revealed a block in activation of DENV-infected moDCs that was not observed in bulk culture (50, 59). Similarly, non-infected bystander monocytes, moDCs and cDCs, expressed higher levels of CD80 and CD86 than DENV-infected cells in the dermis of intradermally infected *Ifnar*^−/−^ mice (53). In addition, DCs produce cytokines and chemokines to modulate T-cell responses. DENV-exposed moDCs (50, 59, 116) or cDCs (61), produce IL-6, IL-10, TNFα, and IFN-α. Furthermore, DENV-exposed moDCs secrete CXCL9, CXCL10, and CXCL11 (118) that bind the chemokine receptor CXCR3 and could attract effector and memory T-cells. However, it remains unclear whether it is the DENV-infected or non-infected DCs that produce these inflammatory mediators. Together, these data suggest that DENV blocks activation in infected DCs, which may decrease the priming of CD4^+^ or CD8^+^ T-cells, whereas non-infected bystander cells still can become activated.

Mixed lymphocyte reactions (MLR) are a functional read-out for DC–T-cell interactions, in which DCs from MHC mismatched donors activate allogeneic T-cells. DENV infection decreased the capacity of moDCs (116, 119) as well as DCs isolated from human skin explants (65) to stimulate proliferation of DENV-naïve CD4^+^ T-cells in MLRs *in vitro*, suggesting that DENV-infected DCs are less capable of activating CD4^+^ T-cells (Figure 2B). Others have reported that DENV-infected cultures of moDCs can prime CD4^+^ T-cells, but with decreased T-cell effector functions, such as secretion of IFN-γ or TNFα (109). The impaired ability of DENV-infected moDCs to produce IFN-α and IFN-β may explain the decreased ability to prime T-cell responses (120). Although these surrogate MLR assays are interesting, little information exists as to how DCs prime DENV-specific T-cells. Non-infected moDCs that were pulsed with DENV E protein efficiently activated CD4^+^ or CD8^+^ CD45RO^+^ memory T-cells from DENV-immune but not from naïve individuals to produce IFN-γ (121). More study is needed to determine the capacity of DCs to prime DENV-specific naïve T-cells or to reactivate memory T-cells during re-exposure. Rapid progress should be possible given the publication of large numbers of HLA-restricted immunodominant DENV antigens (29, 31).

Also, activated T-cells can support the maturation of DCs. An initial study showed that co-culture of moDCs with a CD40L-transfected cell line restored the ability of DENV-infected moDCs to induce MLR responses (114) (Figure 2C). Subsequent work demonstrated that co-culture of activated T-cells with DENV-infected moDCs rescued the otherwise suppressed MLR response, and the activation of non-infected bystander moDCs depended on TNFα and IFN-α/β (118). Consequently, stimulation of the adaptive response to DENV infection requires signals from both cells in DC–T-cell interactions, likely via cell-to-cell contact. In clinical studies, gene expression analysis of acute dengue patients revealed that DHF cases expressed lower levels of genes linked to antigen processing, presentation, and T-cell activation compared to DF patients (122). Thus, impaired antigen presentation and functionality of DENV-infected DCs may reflect a viral immune escape strategy to dampen T-cell responses and impact disease severity.

To date, mostly *in vitro*-generated human moDCs have been used to study the role of DCs in inducing innate or adaptive immune responses during DENV infection. Nevertheless, diverse subsets of cDCs in lymphoid or non-lymphoid tissues execute specialized functions in presenting antigen and inducing CD4^+^ or CD8^+^ T-cell responses (123, 124). Expanding previous findings using human cDC subsets directly isolated from blood or tissues will be important to characterize the full spectrum of immune responses to DENV infection.

## Concluding Remarks

Dengue virus infects the same cells (DCs, monocytes, and MΦs) that are essential for inducing and maintaining optimal innate and adaptive immune responses. This tropism of DENV appears to impair DC function, which may undermine the priming of DENV-specific memory responses. Is it possible to block DENV replication in DCs to reduce viral load and restore DC function, which could impact the generation of neutralizing or potentially harmful memory B- and T-cell responses? Can we as a field harness the knowledge gained about DC biology during DENV infection to prevent human disease? Can DC function be optimized in the context of live-attenuated DENV vaccines to stimulate protective immunity?

Significant progress has been made on characterizing DENV infection and activation of DCs, as well as protective or enhancing T-cell and B-cell responses. Nevertheless, early events after DENV transmission in the skin require further study, including a greater understanding of early virus replication, local immune responses at the site of transmission (i.e., the skin), and immunomodulatory effects of mosquito saliva. Further effort should focus on how responses of DCs impact disease outcome in an acute infection and prime immunological memory responses that will affect dengue pathogenesis and disease severity in secondary DENV infections. A collaborative effort using a multi-disciplinary approach among experts in dengue virology, medicine, vector biology, and immunology is called for to reach these goals.

## Conflict of Interest Statement

The authors declare that the research was conducted in the absence of any commercial or financial relationships that could be construed as a potential conflict of interest.
